# New Trends in the Utilization of Intravenous Fluids

**DOI:** 10.7759/cureus.14619

**Published:** 2021-04-21

**Authors:** Mohammad Tinawi

**Affiliations:** 1 Nephrology, Nephrology Specialists, Munster, USA; 2 Internal Medicine, Indiana University School of Medicine Northwest-Gary, Gary, USA

**Keywords:** intravenous fluids, crystalloid solutions, colloid solutions, fluid resuscitation, albumin, hydroxyethyl starch, isotonic saline, lactated ringer, balanced solutions, plasma-lyte

## Abstract

Intravenous fluids (IVFs) are the most commonly used drugs in hospitalized patients. Knowledge of the indications and pharmacokinetics of IVFs is critical for all medical disciplines. Isotonic saline (normal saline, 0.9% NS) is the most utilized intravenous solution. Isotonic saline effectively expands the intravascular compartment, as one-quarter of the infusate goes intravascularly, while the remaining three-quarters go into the interstitial space. The proper use of IVFs in different clinical scenarios is paramount. IVFs differ with regard to their half-life, intravascular volume expansion, preparation, and cost. Crystalloids are more commonly utilized due to their relatively low cost and availability. Colloids are very advantageous in cases of shock or hemorrhage, as they remain in the intravascular space, thus facilitating an increase in blood pressure (BP) prior to blood administration. Colloids are also advantageous in cases of burns and severe hypoglobulinemia. Human albumin (5%, 20%, and 25%) is the most used colloid solution. It remains intravascularly provided and there is no capillary leak as in systematic inflammation. The goal in hospitalized patients is timely and adequate intravenous fluid resuscitation. Utilization of a large volume of isotonic saline may lead to hypervolemia, hypernatremia, hyperchloremia, metabolic acidosis, and hypokalemia. The use of balanced intravenous solutions has been advocated to avoid these complications.

## Introduction and background

This review article focuses on the understanding of the pharmacokinetics and indications of intravenous fluids (IVFs). The emphasis will be on recent clinical trials illustrating new trends in the utilization of IVFs. PubMed database was searched for relevant basic science and clinical articles in addition to the leading journals in nephrology, critical care, and internal medicine. The material reviewed includes clinical trials, comprehensive reviews, as well as chapters from major textbooks.

Isotonic saline or Ringer’s lactate (lactated Ringer, LR) is usually the first choice in patients with volume depletion who are normonatremic. Patients who are volume-depleted and hypernatremic are given hypotonic solutions such as half-normal saline (0.45% NS) or 5% dextrose in water (D5W). Hypotensive patients are resuscitated with isotonic saline or LR irrespective of their sodium, then they are changed to hypotonic solutions once extracellular fluid (ECF) volume is restored and blood pressure (BP) is normalized [1]. Administration of a large volume of isotonic saline may lead to hypernatremia, hyperchloremia, non-anion gap metabolic acidosis, and hypokalemia [2]. After initial resuscitation, many patients will need the addition of potassium chloride (KCL) to the initial intravenous solution. Others develop hypernatremia and will need a change to a more hypotonic intravenous solution. With prolonged administration of isotonic saline, other electrolytes such as magnesium, calcium, and phosphate will need to be replaced. Lactic acidosis is seen in many patients with hypovolemic shock. These patients are usually resuscitated with isotonic saline which improves lactic acidosis, hemodynamics, and oxygenation. In most cases, sodium bicarbonate solutions are not used in resuscitation because they increase carbon dioxide production resulting in intracellular acidosis. Their use is restricted to patients with arterial pH <7.2. Administration of sodium bicarbonate in metabolic acidosis has not been shown to improve survival in any study in humans [3].

Human albumin (5%, 20%, and 25%) and hetastarch (6% hydroxyethyl starch) are examples of colloid-containing solutions. Colloids are large molecules that increase the plasma oncotic pressure and remain in the intravascular compartment unless there is a disruption to the transcapillary barrier as in multisystem organ failure [1, 4, 5]. Colloid solutions are expensive. In the United States, the average cost of 100 ml of isotonic saline, iso-oncotic albumin (4%-5% albumin), and hyperoncotic albumin (20%-25% albumin) is 0.10, 15.91, and 108.75 US dollars respectively [6]. The price can vary among vendors and pharmacies.

The Saline versus Albumin Fluid Evaluation (SAFE) study found no difference in outcome between 4% albumin or isotonic saline for fluid resuscitation in 6997 patients in multicenter medical and surgical intensive care units (ICU) after 28 days [7]. A meta-analysis of 55 studies showed no effect of albumin on mortality when compared to crystalloid solutions in critically ill patients [8]. Observational studies have raised concerns regarding the use of large volumes of isotonic saline for resuscitation. The use of balanced crystalloid solutions has been suggested as an alternative. Balanced solutions are associated with hypoosmolality, hyperkalemia, and metabolic alkalosis. The Saline Against Lactated Ringers or Plasmalyte in the Emergency Department (SALT-ED) trial found no difference in hospital-free days in noncritically ill patients treated in the emergency department (ED) with either saline or balanced crystalloids [9]. 

## Review

Intravenous fluids should be treated as any other drug. The indication and duration of therapy should be clear in the mind of the prescriber. It is essential to know the 4 D’s of IVF therapy: drug selection, dosing, duration, and de-escalation [10]. The main indications for IVFs are fluid resuscitation, replacement, and maintenance [11]. When IVFs are given to resuscitate critically ill patients, such therapy has four phases: resuscitation, optimization, stabilization, and de-escalation (or evacuation) [10, 11]. Review of clinical studies related to IVFs requires identification of the indication of IVFs, the patient population, and the phase of treatment (in case of critically ill patients). Without such knowledge comparison of different studies becomes meaningless. For example, a study in non-critical ED patients is different from a study in critically ill patients with shock. Each indication and phase may require different IVFs. For example, isotonic saline or LR are appropriate for the resuscitation of a patient in septic shock. After the initial phase, a hypotonic solution such as 0.45 NS may be needed due to hypernatremia. In the de-escalation phase, the same patient may require discontinuation of IVFs and the use of diuretics to relieve hypervolemia. Patients who are unresponsive to diuretic therapy will require renal replacement therapy (RRT). Analysis of the Program to Improve Care in Acute Renal Disease (PICARD) study revealed that critically ill patients with fluid overload and acute kidney injury (AKI) are significantly less likely to recover kidney function [12]. In this study, fluid overload was defined as >10% accumulation. Fluid overload at the time of AKI diagnosis and RRT initiation increased mortality at 30 days (37 vs. 25%, P=0.02) and 60 days (46 vs. 32%, P=0.006). A retrospective cohort study also found increased mortality in septic patients with fluid overload with and without AKI and chronic kidney disease (CKD) [13].

Composition of common intravenous solutions

Plasma osmolarity is a measurement of different solutes that exist in the plasma [14]. The normal range in adults is 280-295 mOsm/L. While osmolarity refers to the number of osmoles in 1 L of a solution, osmolality refers to the number of osmoles per kg of water (mOsm/kg H_2_O). Oftentimes, the two terms are used interchangeably. Normal plasma sodium is around 140 mEq/L. Since plasma is 93% water and 7% protein and lipids, normal sodium in plasma water is 140/0.93 or approximately 151 mEq/L. 

Intravenous solutions are divided into crystalloids and colloids. Crystalloids can be isotonic, hypertonic, or hypotonic relative to the plasma. An isotonic solution is a solution with the same osmolality as body fluids. Tonicity is effective osmolality. For example, urea is not an effective osmolyte due to its inability to exert osmotic force because it enters the cells freely [15]. A significantly hypotonic IV solution can lead to lysis of red blood cells, this why 0.225% NaCl is given in 5% dextrose. Commonly used intravenous solutions are listed in Table 1.

**Table 1:** Commonly used intravenous solutions

Crystalloid solutions	Colloid solutions
Dextrose in water (5% or D5W, 10%)	Human Albumin (4%, 5%, 20%, 25%)
Sodium Chloride (NaCl) (0.45%, 0.9%, 3%)	Hydroxyethyl starch (HES) (Hetastarch 5%, 6%)
NaCl and D5 solutions (D5 0.225 NaCl, D5 0.45 NaCl, D5 0.9 NaCl),	Dextran 40 and 70
Ringer’s lactate (LR)	
Plasma-Lyte A	

The composition of commonly used crystalloid solutions is summarized in Table 2 [2, 11].

**Table 2:** The composition of commonly used crystalloid solutions. The concentration of sodium, chloride, potassium, calcium and lactate is in mEq/L. Na+ (sodium), Cl- (chloride), K+ (potassium), Ca2+ (Calcium). *Plasma-Lyte A contains 27 mEq/L acetate, 23 mEq/L gluconate, and 3 mEq/L magnesium.

Solution	Calculated Osmolarity mOsm/L	Na^+^	Cl^-^	K^+^	Ca^2+^	Lactate	Glucose g/L
D5W	278	0	0	0	0	0	50
0.9 NaCl	308	154	154	0	0	0	0
0.45 NaCl	154	77	77	0	0	0	0
D5 0.9 NaCl	586	154	154	0	0	0	50
3% NaCl	1026	513	513	0	0	0	0
Ringer’s Lactate	272	130	109	4	3	28	0
Plasma-Lyte A	294	140	98	5	0	*	0

The composition of commonly used colloid solutions is summarized in Table 3 [2, 11].

**Table 3:** The composition of commonly used colloid solutions. The concentration of sodium and chloride is in mEq/L. Note that the above solutions have the same concentration of sodium and chloride as isotonic saline. HES: hydroxyethyl starch.

Solution	Calculated Osmolarity mOsm/L	Na^+^	Cl^-^	Albumin g/L	HES g/L
Albumin 5%	308	154	154	50	0
Albumin 25%	308	154	154	250	0
Hetastarch 6%	310	154	154	0	60

Crystalloid solutions

The half-life of crystalloid intravenous solutions is 20-40 minutes in conscious humans. In the presence of dehydration or preoperative stress, such half-life may become 80 minutes or longer [16]. Studies in volunteers showed a longer half-life of isotonic saline compared to Ringer’s lactate [17].

Dextrose in Water

**Figure 1:**
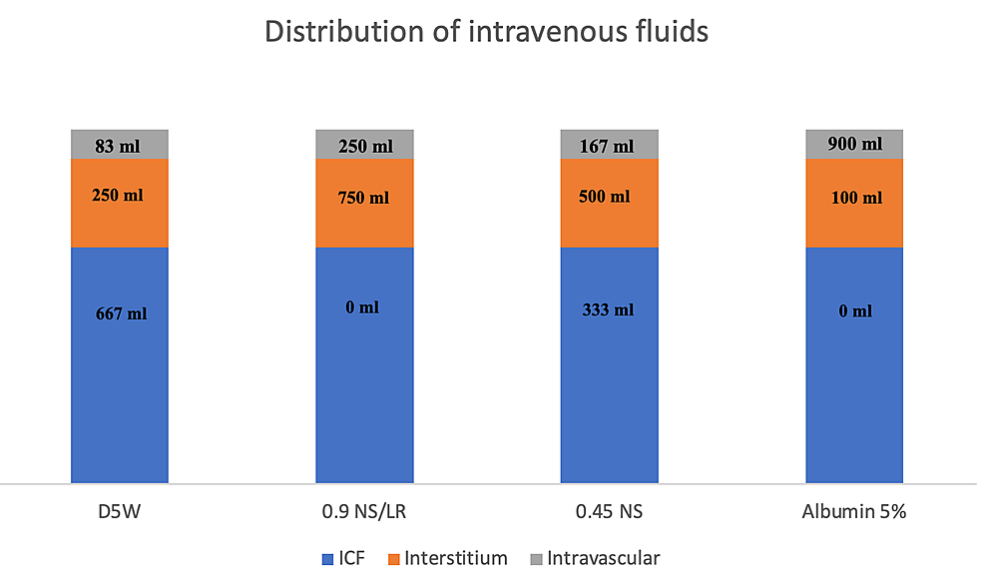
Comparison of the distribution of 1 L of common IVFs in a lean 80 kg man, assuming no capillary leak. IVF: intravenous fluid; D5W: 5% dextrose in water; NS: normal saline; LR: Ringer's lactate; ICF: intracellular fluid.

The most widely used dextrose in water solution is the 5% solution (D5W). Its measured osmolarity is 278 mOsm/L and it provides 170 kcal/L. It is metabolized into water and CO_2_. Its distribution matches the water distribution in the ECF and intracellular fluid (ICF) [4]. D5W is used to correct hypernatremia and hypoglycemia. Other dextrose in water solutions such as 10% and 50% are used in the management of severe hypoglycemia. Dextrose in water solutions should not be solely used in patients requiring fluid resuscitation due to hypovolemia, hypotension, shock, or metabolic alkalosis. Dextrose in water solutions should be avoided in patients with hyponatremia. Administration of such solutions to hospitalized patients is the most common cause of inpatient hyponatremia [18]. Hyponatremia may be life-threatening especially in post-operative female patients due to their tendency to develop the syndrome of inappropriate secretion of anti-diuretic hormone (SIADH) [19]. Now let us take for example a lean 80 kg man: his total body water (TBW) is approximately 60% of his weight. One-third of TBW is in the ECF and 2/3 are in the ICF. Of the 1/3 in the ECF, ¼ (8.3% of TBW) is intravascular, and ¾ (25% of TBW) is in the Interstitium. If 1 L of D5W is administered, only 83 ml goes to the intravascular compartment, making this solution a poor choice for patients with hypotension or hypovolemic shock as indicated above [1]. Figure 1 compares the distribution of 1 L of common intravenous solutions in different body compartments of a lean 80 kg man.

Isotonic Saline (0.9% NS, 0.9 NaCl, Normal Saline)

Isotonic saline is the most commonly used crystalloid solution with over 200 million liters administered in the United States annually [2]. The pH of isotonic saline is around 5.60, which is acidic. Pure distilled water at 25° has a neutral pH of 7. Carbon dioxide (CO_2_) is absorbed into isotonic saline solution upon contact with the atmosphere which subsequently lowers its pH to around 5.60. It is well known that infusion of large amounts of isotonic saline results in normal anion gap metabolic acidosis. This is not caused by the acidic pH of such solution, rather, it is due to dilution of the endogenous buffer systems in the plasma (HCO_3_^-^/ CO_2_) [20]. Moreover, isotonic saline leads to volume expansion and a decrease in proximal HCO_3_^-^ reabsorption. Another contributor is an increase in the activity Cl^-^/HCO_3_^-^ exchanger (pendrin) in beta-intercalated cells in the collecting duct of the nephron. Unlike Ringer’s lactate, isotonic saline has no buffer base. The chloride content of isotonic saline is high compared to plasma (154 mEq/L in isotonic saline vs. 98-106 mEq/L in plasma). Isotonic saline is commonly used to expand ECF volume in hypovolemia, and to treat hypotension, chloride-sensitive metabolic alkalosis, and hyponatremia associated with hypovolemia [15, 21, 22]. Infusion of 1 L of isotonic saline expands the intravascular compartment by 250 ml, making this solution a good choice for patients requiring fluid resuscitation as in sepsis [1].

0.45 Saline (0.45% NaCl, Half-Normal Saline)

One liter of 0.45 saline is equivalent to 500 ml of water added to 500 ml of isotonic saline [15]. When 1 L of 0.45 saline is given intravenously 167 ml goes into the intravascular compartment (Figure 1). This solution is inappropriate for initial resuscitation but is frequently used in the optimization and stabilization phases of fluid therapy. 0.45 NaCl is also helpful in the management of hypernatremia because it provides free water [1].

Ringer’s Lactate (Lactated Ringer, LR)

Ringer’s lactate is a buffered crystalloid solution because it contains sodium lactate [2, 11]. It does not have lactic acid; therefore, it does not cause lactic acidosis. LR is appropriate for initial resuscitation as is isotonic saline. It is slightly hypotonic compared to isotonic saline (LR calculated osmolality is 272 mOsm/L vs. 308 mOsm/L for isotonic saline). Isotonic saline is therefore preferred in the management of hyponatremia, chloride-sensitive metabolic alkalosis, and in patients with hyperkalemia. LR should be used with caution in patients with hepatic failure who may not be able to metabolize lactate to bicarbonate. LR is a balanced electrolyte solution. It contains a buffer (lactate), potassium, and calcium. Moreover, LR has a lower chloride content making it more physiologically balanced. Further advantages of LR based on recent clinical trials are discussed below.

Plasma-Lyte A

Plasma-Lyte A is a balanced electrolyte solution with a pH of 7.4 [2, 15]. Its osmolarity is close to that of isotonic saline; however, its chloride content is significantly lower. It contains potassium and magnesium. Plasma-Lyte A contains 27 mEq/L acetate and 23 mEq/L gluconate. Plasma-Lyte A is appropriate for initial fluid resuscitation and is considered more physiologic compared to isotonic saline [1]. It is not appropriate for patients with chloride-sensitive metabolic alkalosis or hyperkalemia.

Isotonic Saline versus Physiologically Balanced Crystalloid Solutions

Several observational studies have raised concerns about isotonic saline due to its high chloride content and its ability to result in non-anion gap hyperchloremic metabolic acidosis when used in large quantities. Shaw et al. conducted a retrospective observational study in approximately 30,000 patients undergoing major abdominal surgery. In-hospital mortality was 2.9% in the group who received Plasma-Lyte A and 5.6% in the isotonic saline group (P< 0.001) [23]. Complications were also more common in the isotonic saline group including AKI requiring RRT, postoperative infections, acidosis, and blood transfusions. Another observational study performed a retrospective analysis of approximately 110,000 patients with severe inflammatory response syndrome (SIRS) who received fluid resuscitation with crystalloids. The study examined the association between changes in serum chloride concentration and the administered chloride load with in-hospital mortality. After adjustment for severity of illness, mortality increased with increasing chloride load in administered IVFs (≥ 105 mEq/L) (odds ratio OR 1.094, 95% Confidence Interval CI, 1.062 to 1.127) [24]. Krajewski et al. did a meta-analysis of 21 studies involving 6235 critically ill or surgical patients who received either high-chloride (>111 mEq/L) or low-chloride (111 mEq/L or less) crystalloids for resuscitation. There was no difference in mortality based on chloride content; however, there was a significant association between high chloride content crystalloids and several unfavorable outcomes including AKI, hyperchloremic metabolic acidosis, and time on mechanical ventilation [25]. Other studies have also shown improved clinical outcomes (reduced incidence of AKI and lower mortality) in critically ill patients receiving balanced solutions compared to those receiving isotonic saline [26-27].

Two recent randomized trials evaluated balanced crystalloids versus isotonic saline in critically and non-critically ill patients. Both trials were conducted concurrently at Vanderbilt University Medical Center in Nashville, Tennessee, USA. The Saline against Lactated Ringer’s or Plasma-Lyte A in the Emergency Department (SALT-ED) trial was conducted in non-critically ill adults given intravenous crystalloid in the ED before hospital admission outside the ICU [9]. SALT-ED was a pragmatic, single-center, unblinded, and multiple-crossover trial. The trial enrolled 13,347 adult patients and compared isotonic saline to LR or Plasma-Lyte A. Median fluid volume was approximately 1 L per patient. There was no difference in hospital-free days (days alive post-discharge before day 28) between the two groups. Balanced crystalloids solutions were associated with a lower incidence of major adverse kidney events within 30 days (MAKE30) (4.7% vs. 5.6%, adjusted OR; 0.82, 95% CI, 0.70 to 0.95; P=0.01). Patients with serum creatinine equal to or greater than 1.5 mg/dl had a greater benefit from balanced solutions. Most patients in the balanced crystalloid group received LR (95.3%) and only 4.7% received Plasma-Lyte A. The components of MAKE30 were death, RRT, and persistent renal dysfunction (doubling of serum creatinine from baseline).

The Isotonic Solutions and Major Adverse Renal Events Trial (SMART), was a pragmatic, cluster-randomized, multiple-crossover trial in 15,802 adult patients admitted to medical (SMART-MED) and nonmedical (SMART-SURG) ICUs in a single center [28]. The trial compared isotonic saline to LR or Plasma-Lyte A. The primary outcome was MAKE30 (similar to SALT-ED trial). MAKE30 was higher in the isotonic saline group (marginal OR, 0.91; 95% CI, 0.84 to 0.99; conditional OR 0.90; 95% CI, 0.82 to 0.99; P=0.04). In-hospital mortality at 30 days was higher in the saline group 11.1% vs. 10.3% in the balanced-crystalloids, but the difference did not reach statistical significance (P=0.06). The advantage of a balanced solution was more pronounced in the subgroup of patients with sepsis (MAKE30 OR, 0.80; 95% CI, 0.67 to 0.94; P=0.01). Median fluid volume was approximately 1 L per patient in the first 7 days (range 0-3.5L). Most patients in the balanced crystalloid group received LR (91%). 

Both SALT-ED and SMART were unblinded single-center trials, data were censored at the time of discharge, and the treating clinicians determined when to initiate RRT. Over 90% of patients in the balanced crystalloid group received LR, which in essence makes both trials a comparison of isotonic saline to LR. The volume of fluids used for resuscitation was modest.

Two ongoing clinical trials are comparing isotonic saline to Plasma-Lyte A, the Balanced Solution Versus Saline in Intensive Care Study (BaSICS), NCT02875873, which is being conducted in Brazil; and the Plasma-Lyte 148 versUs Saline Study (PLUS), NCT02721654, which is being conducted in Australia/New Zealand.

Colloid solutions

Colloid solutions are more expensive and less widely available than crystalloid solutions. Examples of colloid solutions include human albumin, hydroxyethyl starch (HES), dextrans, and gelatins. Dextran is available as dextran-40 and dextran-70. Dextrans and gelatins are not commonly used and have been rarely associated with anaphylactoid reactions [29]. Severe anaphylactic reactions have occurred in patients with extremely high titers of dextran-reactive antibodies. Anaphylactic reactions to gelatin-based solutions are uncommon, but are usually severe, and can present with latency up to 10-70 minutes post-administration. 

Human Albumin

Albumin 5%, albumin 20%, and albumin 25% are the most used colloid solutions. Also available is 4% albumin solution. Human albumin 20%, Behring, is a low-salt albumin because it contains 125 mEq of sodium/L vs. 154 mEq sodium/L for other albumin solutions. Albumin essentially remains in the intravascular compartment allowing utilization of a lesser volume of fluid to achieve resuscitation [30]. Albumin molecular weight is 69 kD. Albumin increases oncotic pressure in the plasma and has a half-life of 16-24 hours. Patients should be monitored for allergic reactions. The maximum volume expansion of 5% albumin is around 90% (of administered volume). Albumin administration results in an oncotic action attracting 18 ml of water per 1 g of albumin [31]. For 25% albumin the maximum volume expansion is 300%-500% [30]. A 100 ml vial of 25% albumin can expand the intravascular compartment by approximately 450 ml, which is equivalent to 1.8 L of isotonic saline or LR. Normally, 12.5-25 g (250-500 ml) of 5% albumin are given over 30 minutes, while 25 g (100 ml) of 25% albumin are given over 2 hours. 

International guidelines recommend albumin infusion in patients with hepatorenal syndrome, spontaneous bacterial peritonitis, and post large-volume paracentesis due to its anti-inflammatory effect [32, 33]. 25% albumin is commonly given after large-volume paracentesis at a dose of 5-10 g per liter removed [34]. Albumin infusion was recently investigated in a randomized, multicenter, open-label trial involving 777 patients hospitalized with decompensated cirrhosis [35]. On admission, patients had serum albumin <3 g/dl. The primary endpoint was a composite of infection, renal dysfunction, or death between days 3-15 post-hospitalization. The albumin group received on average 143 g of albumin more than the standard care group. The trial concluded that albumin infusion to increase serum albumin level to at least 3 g/dl had no benefit in patients hospitalized with decompensated cirrhosis.

The above-mentioned Saline versus Albumin Fluid Evaluation (SAFE) study demonstrated the safety of intravenous iso-osmolar (4%) albumin in the resuscitation of critically ill patients, while at the same time showing no advantage of 4% albumin over isotonic saline [7]. In the SAFE trial, 6997 critically ill patients were randomized to 4% albumin or isotonic saline for intravenous resuscitation. There was no difference between the two groups in the primary outcome which was all-cause mortality at 28 days. Moreover, there was no difference in the need for RRT, length of stay in the ICU, or days on mechanical ventilation. In a prespecified subgroup analysis of patients with severe sepsis in the SAFE trial, the use of 4% albumin was associated with a lower adjusted risk of death compared with isotonic saline [36]. The same finding was supported in a meta-analysis [37].

Albumin should be avoided in patients with traumatic brain injury. A post-hoc analysis of the SAFE trial involving 460 patients showed that critically ill patients with traumatic brain injury had higher mortality rates when resuscitated with albumin (relative risk RR, 1.63; 95% CI, 1.27 to 2.26; P=0.003) [38]. Patients on intracranial pressure monitoring had an increase in intracranial pressure.

The CRISTAL (Colloids Versus Crystalloids for the Resuscitation of the Critically Ill) study was a multicenter international study conducted over nine years in 57 ICUs [39]. A total of 2857 critically ill patients presenting with hypovolemic shock were randomized to receive colloids (HES, gelatins, dextrans or 4% or 20% albumin) or crystalloids (Ringer’s lactate or isotonic or hypertonic saline). There was no significant difference between the two groups in 28-day mortality. Patients receiving colloids had lower 90-day mortality (30.7% vs. 34.2%; RR, 0.92; 95% CI, 0.86 to 0.99; P=0.03). The authors emphasized that this finding should be considered exploratory and further studies are needed.

The ALBIOS (Albumin Italian Outcome Sepsis) study was an open-label, multicenter study in 1818 ICU patients with severe sepsis. Patients were randomized to 20% albumin and a crystalloid or crystalloid alone. Target serum albumin in the albumin group was equal to or greater than 3 g/dl. In the first seven days of the trial, patients in the albumin group had a lower net fluid balance (P<0.001) and a higher mean arterial pressure (P=0.03) compared to the crystalloid group. There was no difference in the rate of survival at 28 and 90 days, and no difference in the length of stay in the ICU and hospital [40].

Albumin is widely used for the treatment and prevention of intradialytic hypotension. Clinical trials regarding the effectiveness of albumin in hemodialysis patients have been generally small, single-center, and heterogeneous with regard to the intervention [6].

Some observational studies have suggested an increased risk of AKI with hyperoncotic albumin [41, 42]. A recent randomized study in 220 patients undergoing off-pump coronary artery bypass surgery arrived at the opposite conclusion. Patients with albumin less than 4 g/dl were given 20% hyperoncotic albumin or the same volume of isotonic saline preoperatively. Patients in the hyperoncotic albumin had less postoperative AKI (13.7% vs. 25.7%, P=0.048) [43]. A meta-analysis of 10 studies involving 6664 patients with sepsis showed no increase in the use of RRT with albumin when compared to crystalloids [44].

Hydroxyethyl Starch (HES)

HES is a synthetic colloid. HES as in the case of dextrans and gelatins has been associated with anaphylactoid reactions [29]. Hetastarch is the most used HES and is available in 3%, 5%, 6%, and 10% solution. Its average molecular weight is 450 kD. Hetastarch has a calculated osmolarity around 310 mOsm/L and a long half-life of 50 hours. Its maximum volume expansion is 100-200% [30]. Randomized clinical trials have shown an increased need for RRT and higher mortality in critically ill patients treated with HES when compared to either isotonic saline or Ringer’s acetate [45, 46]. A meta-analysis of 38 trials concluded that acute volume resuscitation in critically ill patients with HES was associated with an increased risk of mortality and AKI when compared with other fluids [47]. The US Food and Drug Administration (FDA) warned against the use of HES in critically ill patients due to an increased risk of AKI and death. HES solutions such as Hespan® (6% hetastarch in 0.9% sodium chloride) (B. Braun Medical Inc., Bethlehem, PA, USA) have a black box warning [48]. More recently, the Fluid Loading in Abdominal Surgery: Saline vs Hydroxyethyl Starch (FLASH) trial was published [49]. FLASH was a multi-center, double-blind, randomized trial in 775 patients undergoing major abdominal surgery and at risk for postoperative AKI. Patients were randomized to receive HES or 0.9% NS using an individualized hemodynamic algorithm starting at the time of surgery and for up to 24 hours postoperatively. At 14 days postoperatively, there was no significant difference in the primary outcome which was a composite of major postoperative complications or death. At 28 days postoperatively, AKI had occurred in 23% of patients in the HES group and 17% of patients in the saline group (RR, 1.36; 95% CI, 1.02-1.82; P = .04). Based on these findings, there is no justification for the use of HES in this patient’s population.

## Conclusions

The majority of hospitalized patients are given intravenous fluids. Understanding the indications, pharmacokinetics, risks, and benefits of these drugs is essential to all clinicians. The use of a large volume of isotonic saline in the resuscitation of critically ill patients should be avoided unless there is a clear indication such as chloride-sensitive metabolic alkalosis or hyponatremia. Administration of balanced intravenous solutions in the resuscitation of ED, and critically ill patients especially with sepsis may prevent or mitigate AKI. HES preparations should be avoided in critically ill patients due to increased mortality and incidence of AKI. Isotonic albumin is safe for the resuscitation of critically ill patients. Overall, it has no advantage over crystalloids except possibly in patients with sepsis. Albumin administration should be avoided in patients with traumatic brain injury. Ongoing studies will clarify the role of balanced crystalloid solutions. Most of the studies presented in this review have been limited to ED, surgical, or critically ill patients. Further studies are needed in other types of hospitalized patients.
